# Face pareidolia in male schizophrenia

**DOI:** 10.1038/s41537-022-00315-y

**Published:** 2022-12-14

**Authors:** Valentina Romagnano, Alexander N. Sokolov, Patrick Steinwand, Andreas J. Fallgatter, Marina A. Pavlova

**Affiliations:** Department of Psychiatry and Psychotherapy, Medical School and University Hospital, Eberhard Karls University of Tübingen, and Tübingen Center for Mental Health (TüCMH), Tübingen, Germany

**Keywords:** Schizophrenia, Human behaviour

## Abstract

Faces are valuable signals for efficient social interaction. Yet, social cognition including the sensitivity to a coarse face scheme may be deviant in schizophrenia (SZ). Tuning to faces in non-face images such as shadows, grilled toasts, or ink blots is termed *face pareidolia*. This phenomenon is poorly investigated in SZ. Here face tuning was assessed in 44 male participants with SZ and person-by-person matched controls by using recently created Face-n-Thing images (photographs of non-face objects to a varying degree resembling a face). The advantage of these images is that single components do not automatically trigger face processing. Participants were administered a set of images with upright and inverted (180° in the image plane) orientation. In a two-alternative forced-choice paradigm, they had to indicate whether an image resembled a face. The findings showed that: (i) With upright orientation, SZ patients exhibited deficits in face tuning: they provided much fewer face responses than controls. (ii) Inversion generally hindered face pareidolia. However, while in neurotypical males, inversion led to a drastic drop in face impression, in SZ, the impact of orientation was reduced. (iii) Finally, in accord with the signal detection theory analysis, the sensitivity index (*d-prime*) was lower in SZ, whereas no difference occurred in decision criterion. The outcome suggests altered face pareidolia in SZ is caused by lower face sensitivity rather than by alterations in cognitive bias. Comparison of these findings with earlier evidence confirms that tuning to social signals is lower in SZ, and warrants tailored brain imaging research.

## Introduction

Mental disorders are often characterized by impairments in visual social cognition. Reading of bodies and faces is crucial for non-verbal interaction constituting a core of social competence^1–7^. Impairments of this ability lead to misinterpretation of non-verbal signals and, therefore, to inefficient interpersonal interactions and altered mental well-being at large. Social cognition has been shown to be affected in individuals with schizophrenia (SZ), and these deficits may compromise their integration in the society, social participation, and quality of life^8–15^.

SZ represents a mostly chronic disorder with a heterogeneous genetic and neurobiological background^16^. Although SZ is a widely investigated mental condition, understanding of this disease still leaves many open issues^17–22^. The lifetime prevalence of SZ is about 4:1000 individuals, with a lifetime morbid risk of 7.2 per 1000^23^, and the global age-standardized point prevalence of 0.28%^24^. As most neuropsychiatric conditions, SZ is gender/sex-specific disorder possessing a skewed sex ratio: males are more often affected with a ratio ranging from 1.4 to 1.6^22,23,25^. Gender/sex differences occur both in neurobiological correlates and clinical manifestation: age of onset, symptoms, and severity differ between females and males, with an earlier age of onset, worse premorbid functioning, and a greater severity of negative symptoms for males^20,22,26–29^. SZ patients often show cognitive impairments^30^, in particular, mild to severe deficits in social cognition^15^. They exhibit difficulties on the Reading the Mind in the Eyes Test^7,31,32^ and body language reading^10,15,33–41^. Males and females possess distinct profiles in social cognition and metacognition^42–44^. Individuals with SZ display lower sensitivity to faces expressing negative emotions as well as to happy faces, misinterpreting them as negative^45–48^. Yet women with SZ perform generally better on emotion recognition tasks^49,50^. SZ patients not only exhibit aberrant facial affect recognition, but also deficient face processing such as face recognition, visual scanning of faces, and unfamiliar face matching^51–54^. Deficits appear to refer to assessment of the spatial relationship between facial features as well as holistic face perception^55–59^.

**Fig. 1:**
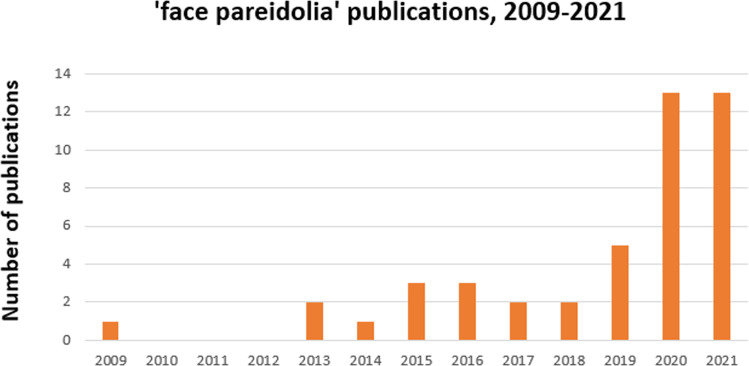
Number of publications on face pareidolia per year between 2009 and 2021. The outcome of PUBMED search with keywords ‘*face pareidolia’* indicates sharply increased interest to the topic over the last years.

So far, a large body of work on face processing has been done with photographs. However, in such images, the mere occurrence of typical features (such as a nose) already implicates face presence. Yet faces can be seen in non-face images such as grilled toasts or clouds^60–65^. This phenomenon reflects tuning to faces termed *face pareidolia* (from the ancient Greek παρά (para)—‘next to it’ and είδωλον (eidolon)—‘shape, image’). In recent years, face pareidolia in face-like non-face images elicits great interest^66–78^ (Fig. 1), primarily because their components do not automatically trigger face processing and, therefore, allow for a proper investigation of face tuning.

To date, a handful of studies on face pareidolia are available in SZ, and the outcome is controversial. It is shown that patients with SZ see face-like objects faster than non-face-like images^79^. In contrast, patients are reported either to identify fewer face-like images as faces^80^ or exhibit higher face pareidolia scores^81^. By presenting Face-n-Food images slightly bordering on Giuseppe Arcimboldo’s portraits (consisting of genius face-resembling food compositions^82–88^) in ascending order from the least to most face resembling, higher face thresholds were found in SZ^89^.

**Fig. 2:**
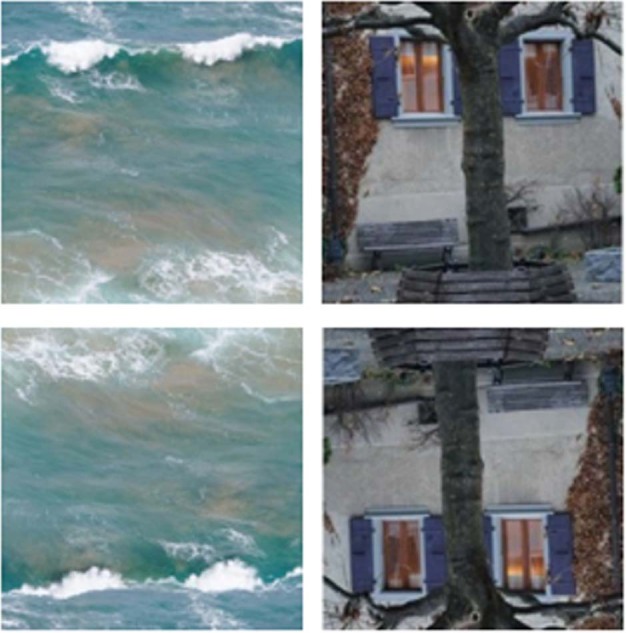
Examples of the Face-n-Thing images with canonical upright (top) and inverted (bottom) display orientation. The image on the left is an example of one of the least face resembling, and the image on the right is one of the most face resembling when presented with upright orientation. The images in the left column (‘waves’) had been first published in Pavlova et al. 2020 PLoS ONE; the Creative Commons Attribution [CC BY] license.

It is well-known that face perception is hindered by display inversion in the image plane (*face inversion effect*, FIE)^90–92^. Display inversion provides a proper control for face tuning since the overall amount of intra-stimulus information is the same with both orientations. It is widely believed that in full-seen faces, display inversion affects holistic face perception^93^. Although previous work with face-like images used inverted displays^94–96^, it was taken for granted that inversion deteriorates face impression in such images. Our earlier study^62^ showed that face pareidolia in non-face face-like images presented upside-down (Fig. 2) dropped for about 25% in neurotypical females and for almost 50% in males.

The present study was aimed at investigation of face tuning in a rather homogeneous sample of males with SZ. We examined (i) whether SZ patients demonstrate lower sensitivity to face-like images; and (ii) whether, and, if so, how display inversion affects face tuning.

## Methods

### Participants

Forty-four participants were enrolled in the study. Twenty-two patients with SZ aged between 21 and 45 years were recruited from the inpatient units at the Department of Psychiatry and Psychotherapy, University Hospital, Eberhard Karls University of Tübingen, Germany. The sample size was determined prior to the study by demands of statistical data processing, and was calculated taking into account possible dropouts. Twenty-one patients were diagnosed with paranoid SZ (ICD-10, F20.0); for one patient, the diagnosis had changed in the course of hospitalization to schizoaffective disorder (ICD-10, F25). Average time from the first diagnosis was 8.85 ± 7.90 years (mean ± standard deviation, SD; median, Mdn, 6.5 years; 95% confidence interval, CI [5.15; 12.55]). Duration of hospitalization before examination was 39.14 ± 66.22 days (Mdn, 12 days; 95% CI [9.00; 69.29]). Exclusion criteria comprised preterm birth (<37 gestation weeks) and comorbid psychiatric conditions such as attention deficit hyperactivity disorder and anxiety disorders. In 11 out of 22 patients, self-reported acoustic hallucinations had been documented. None reported experience of visual hallucinations. Ten out of 22 patients had comorbidity: primarily multiple substances abuse disorder/drugs addiction (ICD-10, F19.10; 7 patients), either in combination or not with antisocial personality disorder (F60.2; 1), borderline personality disorder (F60.3; 1), combined personality disorder (F61; 1), post-traumatic stress disorder (F43.1; 1), and cannabis addiction (F12.9; 1). Most patients had a pre-history of drugs intake [cannabis (7) and opioids (3)], as well as alcohol (6) or nicotine (19) consumption. All patients were under routine medical drug treatment: antipsychotics [olanzapine (7); quietiapine (6); risperidone (6); aripiprazole (4); haloperidol (4); clozapine (3)], sedatives [lorazepam (2)], and antidepressants [citalopram (1); bupropion (1)]. The data sets of 22 typically developing (TD) individuals, person-by-person matched for gender and age with SZ patients, were used as a control. None of them had a history of neurological or psychiatric disorders including SZ, autism spectrum disorders (ASD), or major depressive disorder (MDD), and regular intake of medication. TD participants matched to SZ individuals were either recruited from the local community (10 out of 22, 45.45%) or their data sets were taken from our earlier study conducted in TD individuals with the same experimental design^62^.

Data of 44 participants, 22 SZ patients aged 31.41 ± 7.03 years (Mdn, 30.5 years; 95% CI [28.29; 34.53]) and 22 controls aged 30.14 ± 7.03 (Mdn, 28 years, 95% CI [27.02; 33.26]), with no age differences between the groups (Mann–Whitney test, *U* = 214, *p* = 0.516, two-tailed, n.s.) entered the data analysis. All participants had normal or corrected-to-normal vision. The study was conducted in line with the Declaration of Helsinki and approved by the local Ethics Committee at the Medical School, Eberhard Karls University of Tübingen. Informed written consent was obtained from all participants. Participation was voluntary and the data were processed anonymously. Participants received a monetary reward for their participation.

### Task and procedure

The task is described in detail elsewhere^62^. In brief, participants were administered a computer version of the Face-n-Thing task by using Presentation software (Neurobehavioral Systems, Inc., Albany, CA, USA). They were presented with a set of Face-n-Thing images in varying degree resembling a face (Fig. 2). The stimuli subtended a visual angle of 9.8° × 9.8° at an observation distance of 70 cm. The images were presented in pseudo-randomized order, one by one for 1 s with either canonical upright or inverted orientation in three runs. Each experimental session comprised 168 trials (14 images × 2 types [original/mirror image] × 2 display orientations [upright/inverted] × 3 runs). No more than three images with the same orientation appeared consecutively. In this way, a possible visual adaptation to display orientation was prevented. Participants were asked to respond during an inter-stimulus interval. During this interval, a white fixation cross was displayed for duration jittered from 4 to 6 s. If a participant failed to respond within this period, the next trial automatically started. On each trial, in a two-alternative forced-choice (2AFC) task, participants had to indicate by pressing a respective key whether they had an impression of a face. They were explicitly told that there were no correct or incorrect responses, and they had to rely solely upon their own visual impression. Participants were asked to respond as fast as possible after the stimulus offset. No immediate feedback was provided. Instructions were carefully explained to participants and their understanding had been proven with pre-testing (about 10 trials) performed under supervision of an examiner. Participants were run individually. None had previous experience with such stimuli. The testing lasted for about 25–30 min.

### Data processing and analysis

Prior to statistical data processing, all data sets were routinely analyzed for normality of distribution by means of Shapiro–Wilk tests with subsequent use of either parametric (for normally distributed data) or non-parametric statistics. For non-normally distributed data sets, additionally to means and SDs, Mdns and 95% CIs were reported. Inferential statistics was performed by mixed-model analyses of variance, ANOVAs, and post-hoc pairwise comparisons by using Tukey honestly significant difference (HSD) tests with software package JMP (Version 16; SAS Institute, Cary, NC, USA). Non-parametric statistics (Mann–Whitney and Wilcoxon signed-rank tests) was performed for between- and within-group comparisons, respectively, with MATLAB (version 2022a^97^; MathWorks, Inc., Natick, Ma, USA). For calculation of the sensitivity index (*d-prime*) and decision criterion (*beta*) in accord with the signal detection theory (SDT, that helps to measure the ability to differentiate between information-bearing patterns and patterns that distract from the information)^98^, we used MATLAB, version 2022a^97^.

## Results

### Face response rate

A two-way mixed ANOVA was performed on individual face response rates with a within-subject factor Display Orientation (upright/inverted) and between-subject factor Group (patients/controls). Main effect of Display Orientation was significant (*F*(1,42) = 51.36, *p* < 0.0001; effect size, eta squared, *η*^*2*^ = 2.212, with greater face response rates for upright orientation), whereas the effect of Group was non-significant (*F*(1,42) = 0.30, *p* = 0.589; n.s.). The interaction between these factors was significant (*F*(1,42) = 2.74, *p* = 0.001; *η*^*2*^ = 1.102).

**Fig. 3:**
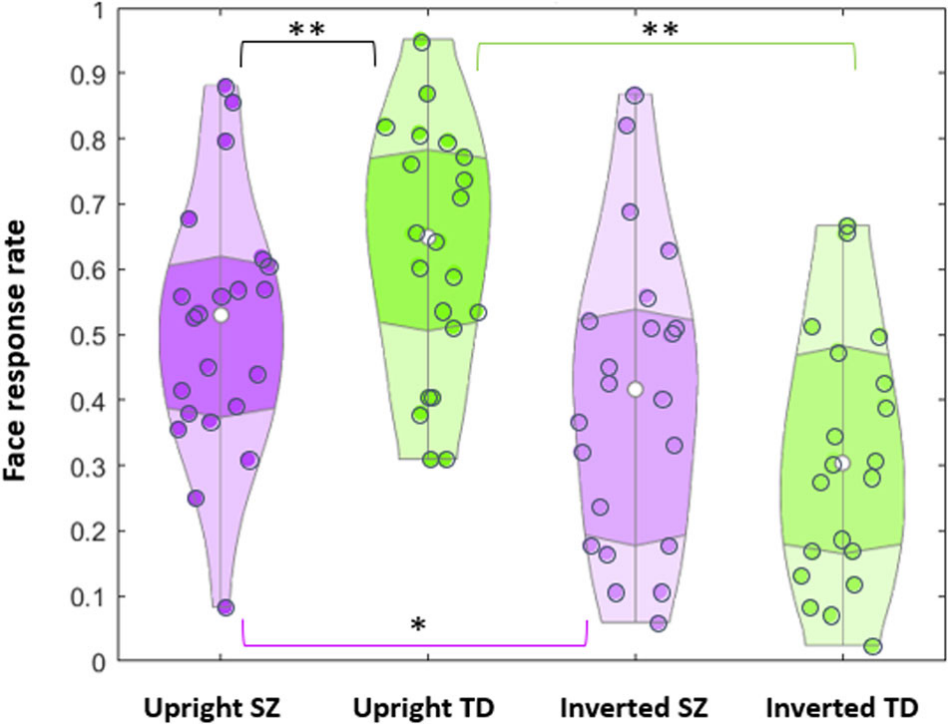
Violin plot of face response rate. Face response rate for SZ patients (violet) and TD controls (green) for upright images on the left and inverted images on the right. White dots within the violins show the mean values. Double asterisks indicate significant differences with upright display orientation between SZ and TD individuals (black line; *p* = 0.028), and effect of inversion for TD individuals (green line; *p* < 0.0001). Single asterisk indicates a tendency of SZ patients to provide fewer face responses with inverted as compared with upright canonical orientation (violet line; *p* = 0.068).

Post-hoc analyses indicated: (i) With upright orientation, face response rate was lower in SZ patients as compared to TD controls (SZ, 0.51 ± 0.19; TD, 0.63 ± 0.19; *t*(42) = 2.91, *p* = 0.028, two-tailed Tukey HSD test throughout, here and further corrected for multiple comparisons; effect size, Cohen’s *d* = 0.668). (ii) With inversion, no difference in face response rate was found between patients and controls (SZ, 0.41 ± 0.23; TD, 0.32 ± 0.20; *t*(42) = 2.14, *p* = 0.158, n.s.). (iii) Display inversion substantially reduced face recognition in TD controls (*t*(21) = 7.59, *p* < 0.0001; effect size, *d*_rm_ = 1.23), but only tended to affect face pareidolia in SZ (*t*(21) = 2.54, *p* = 0.068). In SZ, inversion resulted in a drop of face impression by 20.20%, whereas in TD individuals face impression felt down by 48.80% (Fig. 3).

**Fig. 4:**
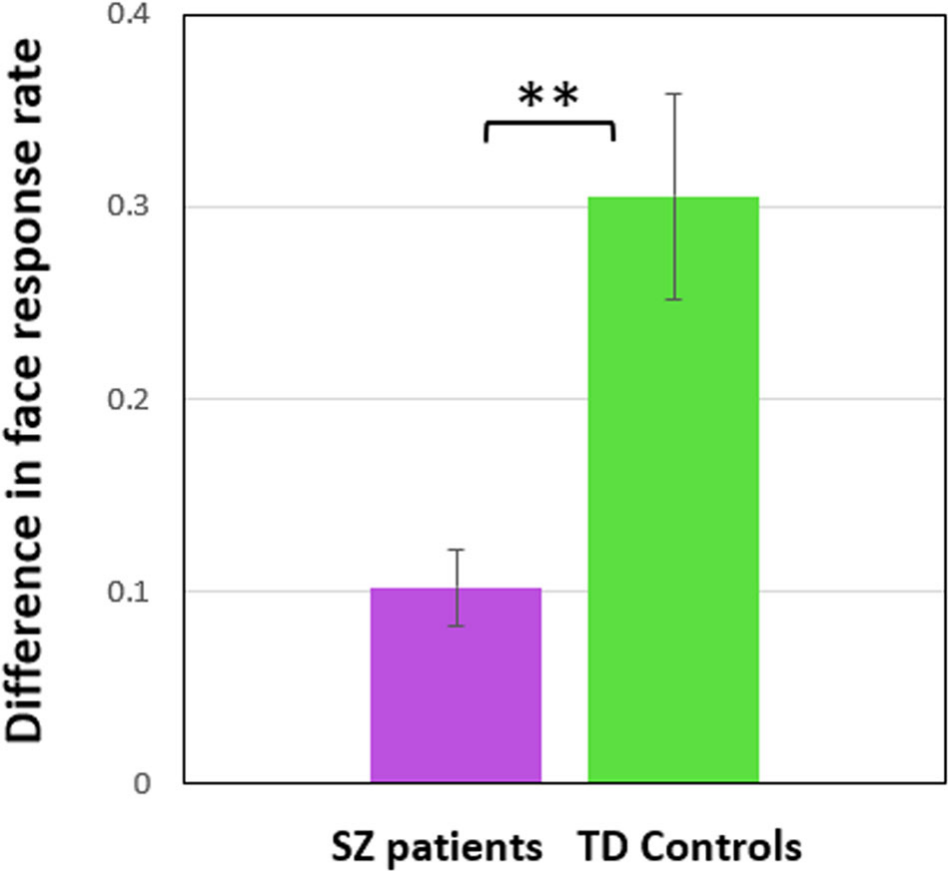
Difference (means) in individual face response rate between upright and inverted orientation. Data for SZ patients are shown in violet, for TD controls in green. Vertical bars indicate ±SEM. Double asterisks show a significant difference (*p* = 0.003) between the groups.

The difference of difference in face response rate with upright and inverted orientation (calculated for each SZ and TD participant) was highly significant (SZ, 0.10 ± 0.09; Mdn, 0.09, 95% CI [0.06; 0.14]; TD, 0.31 ± 0.25; Mdn, 0.21, 95% CI [0.2; 0.42]; Mann–Whitney test, *U* = 114.5, *p* = 0.003, two-tailed; *η*^*2*^ = 0.204; Fig. 4). This underscores that face pareidolia is differentially affected by inversion in SZ and TD individuals.

### Response time

**Fig. 5:**
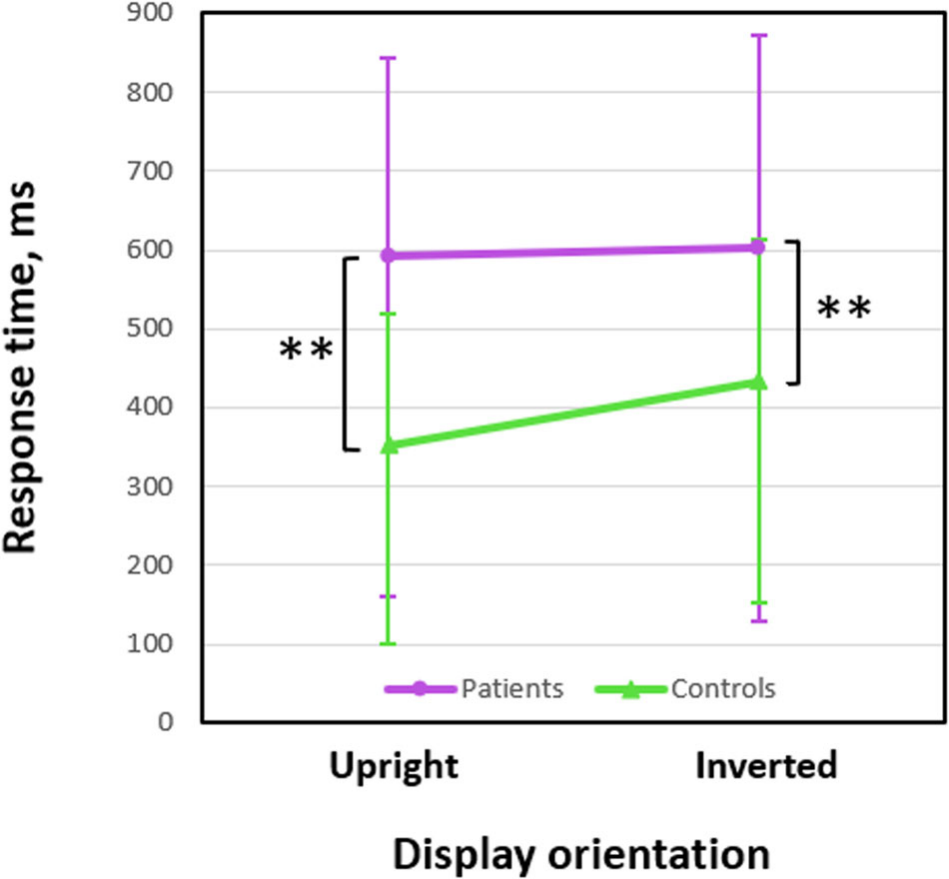
Response time (medians) for the face responses to the Face-n-Thing images. Data for SZ patients are shown in violet, for TD controls in green. Vertical bars represent half 95% CIs. Double asterisks indicate significant differences between SZ patients and TD controls (upright orientation, *p* = 0.009; inverted orientation, *p* = 0.022).

The impact of inversion on response time (RT) was non-significant in SZ patients (upright orientation, 683.44 ± 409.67 ms; Mdn, 593.23 ms, 95% CI [501.81; 865.08]; inversion, 744.77 ± 461.58 ms; Mdn, 602.56 ms; 95% CI [540.12; 949.43]; Wilcoxon signed-rank test, *z* = 0.80, *p* = 0.426, two-tailed, n.s.) as well as in TD controls (upright orientation, 420.41 ± 191.89 ms; Mdn, 352 ms, 95% CI [335.33; 505.49]; inversion, 461.72 ± 227.75 ms; Mdn, 433 ms, 95% CI [360.74; 562.69]; Wilcoxon signed-rank test, *z* = 1.67, *p* = 0.597, two-tailed, n.s.; Fig. 5). With upright orientation (in accord with lower face response rate in SZ), patients also responded slower (Mann–Whitney test, *U* = 130, *p* = 0.009, two-tailed, false discovery rate, FDR, corrected, *η*^*2*^ = 0.157). However, the effect of SZ on RT was also significant with inversion (*U* = 144, *p* = 0.022, two-tailed, FDR corrected; *η*^*2*^ = 0.120). This outcome, therefore, may be considered non-specific in respect to experimental manipulation (changing display orientation): SZ patients responded generally slower than TD individuals with both upright and inverted orientation.

### Visual sensitivity and decision criterion

As poorer performance of SZ patients may have been connected either with lower visual sensitivity to faces in Face-n-Thing images or with more conservative decision criterion (response bias), the SDT analysis^98^ was used to calculate the sensitivity index (*d-prime*) and decision criterion (*beta*). The d-prime was significantly lower in SZ patients (SZ, 0.31 ± 0.30; Mdn, 0.26, 95% CI [0.18; 0.44]; TD, 0.93 ± 0.81; Mdn, 0.55, 95% CI [0.57; 1.29]; Mann–Whitney test, *U* = 121, *p* = 0.005, two-tailed; *η*^*2*^ = 0.183), whereas no difference in decision criterion was found (SZ, 1.16 ± 0.34; Mdn, 1.04, 95% CI [1.01; 1.31]; TD, 1.36 ± 1.17; Mdn, 1.06, 95% CI [0.84; 1.88]; *U* = 230.5, *p* = 0.795, two-tailed, n.s.).

### Face pareidolia and hallucinations

As some researchers expect higher face pareidolia rates in SZ patients who are considered to be prone to hallucinations^79,81^, we compared face response rates in SZ patients who self-reported hallucinations and who did not. With canonical upright orientation, face pareidolia rates did not differ between these sub-groups (with hallucinations, 0.54 ± 0.18; without, 0.48 ± 0.21; *t*(20) = 0.02, *p* = 0.490, two-tailed, n.s.). Similarly, no difference was found with display inversion (with hallucinations, 0.39 ± 0.24; without, 0.43 ± 0.23; *t*(20) = 0.43, *p* = 0.675, n.s.).

## Discussion

The present work was aimed at investigation of face pareidolia in males with SZ. We also explored a potential impact of display orientation on face pareidolia as display inversion substantially impairs face impression^62^, and serves a proper control for face recognition. The findings indicated: (i) With upright orientation, males with SZ exhibited lower face tuning to face-like images. (ii) Furthermore, whereas the visual sensitivity to faces was lower in SZ, no difference in decision criterion occurred between SZ and TD individuals. (iii) Inversion hindered face pareidolia in both TD and SZ individuals. However, while in TD males, inversion led to a drastic reduction (about 50%) in face impression, in SZ, the impact of inversion was less pronounced (20% only), which represented a reduced inversion effect.

### Face tuning in schizophrenia

The finding on lower face tuning in SZ with upright orientation is in good agreement with earlier work of our group^89^, which used a different set of images (Face-n-Food instead of Face-n-Thing images here), different presentation mode (images were presented from the least to most face resembling instead of pseudo-randomization here), different task (a spontaneous recognition/open-end instead of a 2AFC paradigm here), upright-oriented stimuli solely, and a mixed group of patients consisting also of females (instead of a homogeneous group of males here). The specificity of deficits in face pareidolia in SZ was confirmed by our work in MDD (with the same paradigm as in Rolf et al.^89^): neither male nor female patients demonstrated deficient face pareidolia^4,5^. In other words, face tuning is not simply lower in any mental disorder. On the same wavelength, cross-disease research in individuals with ASD^85^, Down syndrome^86^, Williams syndrome^83^, and in adolescents born prematurely^88^ reveals substantial (though rather specific for every single condition) deficits in face tuning. These deficits are characterized by dissimilar condition-specific dynamics^88,89^, which sheds light upon their possible origins.

Previous findings on face pareidolia in SZ are extremely sparse and controversial. Individuals with SZ (in the study coordinated by the Nyíro Gyula National Institute of Psychiatry and Addictions, Budapest; *N* = 50, 32 males/18 females) exhibited higher face pareidolia scores than neurotypical individuals and patients with bipolar disorder^81^. Yet, SZ patients (*N* = 10; females and males) identified fewer face-like images as faces (60 images, 20 of which represented faces, 20 face-like non-faces, and 20 non-faces) than controls^80^. By using breaking continuous flash suppression (b-CFS, a kind of binocular rivalry, when one eye is presented with a stimulus such as a schematic face, while the second eye is administered a series of rapidly changing stimuli) in patients with SZ and schizoaffective disorder (8 males/9 females; Australian Schizophrenia Research Bank), it was shown that similar to neurotypical individuals, patients reported seeing faces faster in face-like than in similar non-face images^79^. The differences in the outcome of these studies may be attributed to methodological issues (visual input, tasks, etc.). For example, b-CFS is thought to address the earliest preconscious stages of visual processing supported by the rapid subcortical face-detection pathways^99^. This method is believed to disentangle perceptual mechanisms from higher-order influences such as cognitive biases^79^. It is assumed that a hard-wired subcortical face detection machinery prioritizes early face detection even at potential costs of false positives^65,100^, and in such a way provides privileged access for face-like images to visual awareness. From this perspective, as SZ and TD individuals are rather similar at earlier stages of encoding face-like images under b-CFS conditions^79^, differences in face pareidolia between them may be considered of the cognitive rather than perceptual origin. However, this assumption appears to contradict our SDT analysis demonstrating that SZ and TD individuals exhibit differences in sensitivity rather than in cognitive decision criteria.

### Hallucinations, perceptual errors, and facial affect

Some researchers assume that face pareidolia should be more pronounced in SZ, as many patients are inclined to hallucinations^79,81^. In our opinion, however, experience of face pareidolia requires an interplay between external visual input (a face scheme) and internal face templates. This interplay makes up an impression of seeing faces. For face hallucinations (when internal, self-generated impressions are misattributed to external sources), appropriate visual input is not required. In line with this, in a non-clinical sample of young adults, levels of schizotypy do not correlate with face pareidolia^101^. Yet high positive schizotypy scores are associated with an increased tendency to perceive meaning in visual noise, i.e., with more liberal cognitive criteria to seeing faces^102^. In young adults, proneness to seeing faces embedded in noise is correlated with vividness of mental imagery^73^. More frequent (face and body) pareidolia and hallucinations are reported in patients with dementia with Lewy bodies (DLB)^103^ and Parkinson’s disease (PD)^104^. In patients with DLB^105^, cholinergic enhancement reduces both the number of pareidolia experiences and severity of visual hallucinations. Positron emission tomography shows that the number of pareidolic impressions in PD patients is correlated with hypometabolism in the bilateral temporal, parietal, and occipital cortices, while the index of visual hallucinations is linked to the left parietal cortex, with an overlapping activation in this region during hallucinations and pareidolia^104^. In the present study, however, face pareidolia rate did not differ between SZ patients with and without hallucinations (albeit acoustic, not visual).

Sometimes face pareidolia is considered a kind of perceptual *error*^63^. Again, if images contain a face scheme, face pareidolia may be hardly classified as an *error* but rather as tuning to the presence of such a scheme in the visual input. Indeed, it has been argued that such a scheme (template) does exist, is evolutionary ancient and has the property of a *supernormal stimulus* in the ethological meaning of the term^106^. Obviously, a greater predisposition for seeing faces can reflect fluctuations in the sensitivity level to a face scheme as well as a shift in decision bias affected by higher expectations or even durable desires to seeing a face, for example, of Jesus in a piece of toast^107^ or Elvis in a potato chip^108^. In line with this, paranormal and religious believers are more prone to face pareidolia than sceptics and non-believers^109^. Accordingly, SDT analyses indicate that believers possess more liberal decision criteria without difference between them and sceptics in the visual sensitivity. Within this framework, less frequent face pareidolia experiences may be caused by lower sensitivity, more conservative decision criterion, or a trading between them^110^.

Finally, it appears plausible to expect that individuals who more often experience face pareidolia also have a more efficient affective system, and both systems communicate with each other. In the same vein, face resemblance in face-like images is positively tied with face likability, though this holds true for female perceivers only^84^. Moreover, the more an image resembles a face the more memorable it is^70^. Face-like non-faces may vary in perceived trustworthiness and dominance^72^. Facial expressions (such as happiness) are reported to be processed in a similar way for both face-like non-faces and real faces presumably sharing a common underlying mechanism^63^. Face-like non-faces are readily perceived as having not only emotional expressions, but also gender and age^65,84^. Yet a tendency to judge face-like non-faces as male rather than female (‘*seeing men everywhere, even in toast*’^111^) contradicts earlier findings: face resemblance in Face-n-Food images is associated with ladylike impression^84^. Recent studies in non-human primates show that among brain regions responsive to face-like images, the amygdala plays a decisive role^112^. Moreover, preference for both real faces and face-like non-faces is eliminated in a monkey with bilateral amygdala lesions^100^. This underscores an interaction between visual processing of a socially significant face pattern and its affective value. Although face pareidolia does not represent a uniquely human phenomenon, it is more pronounced in humans. Pre-school children, first trained to select faces among non-faces, later choose not only real full-seen faces but also face-like non-faces, whereas rhesus monkeys (*Macaca mulatta*) and capuchin monkeys (*Sapajus apella)* do not^64^.

### Display inversion effect in schizophrenia

The attention-grabbing finding of the present study is that the FIE in face-like images is profoundly modulated by mental conditions such as SZ. While in TD males, display inversion leads to lessening of face pareidolia to about 50%, in SZ males, it decreases face impressions only by about 20%. In other words, SZ patients more often report face resemblance in *retort* to the Face-n-Thing images presented upside-down.

As considered earlier^62^, inversion is believed to unfavorably affect holistic representations of a face as a whole, thereby forcing less efficient strategies by serial, rather than parallel, processing^113–119^. The FIE is also frequently explained by the lack of experience: in daily life, people used to see faces with canonical orientation, and consequently they develop a predisposition for this orientation^90,120,121^. This explanation is related to the face-scheme incompatibility model^122^ that refers to early stages of face processing. In accord with this view, inversion merely disrupts the face template such as *two eyes are above a mouth*.

The FIE holds true also for face-like images^62^. However, this effect turned out to be gender-specific: males are much more heavily affected by inversion than females^62^. In the present study, the FIE was also observed in TD males of a wider age range. Keeping in mind that the Face-n-Thing images do not contain any elements signaling face presence, it does not appear unforeseen.

Why is the display inversion effect reduced in SZ patients? Does it suggest that they are less vulnerable to disruption in a face scheme elicited by inversion? The FIE in SZ is poorly documented even with full-seen faces, and the outcome is debatable^123,124^. Several studies report the same FIE magnitude in SZ and TD individuals performing facial identity discrimination, face memory recognition, and facial emotion identification^125–127^. Yet the inversion effect magnitude in emotion discriminability is associated with both positive and negative clinical symptoms: the more severe the symptoms are, the weaker the FIE^126^. On a face part-and-spacing task, inversion equally affects performance of patients with SZ/schizoaffective disorder and controls^128^. On the other hand, on all standardized tests assessing basic face perception skills (identity discrimination, memory for faces, recognition of facial affect) and face detection among distractors, the inversion effect in SZ patients is substantially reduced^58,129,130^. Its magnitude is not related to clinical symptoms^130^. The reduced inversion effect on facial affect recognition is also found in unmedicated/antipsychotic-free SZ patients^131^. Administration of ketamine (that can induce a transient psychosis via its influence on ionotropic N-methyl-d-aspartate, NMDA, receptors, and mimic SZ-like symptoms) in a non-clinical sample leads to elimination of the FIE^132^.

If inversion ultimately impedes holistic face processing, then the FIE should be even more evident in SZ individuals known for their deficient holistic processing^124^. Yet it was not the case in the present study. The other possibility is that SZ patients implement a serial analysis of elements even with upright orientation. Consequently, their face tuning becomes lower (as reflected in our findings). In accord with this, in SZ, the M170 component of magnetoencephalographic (MEG) response even to upright full-seen faces is slackly localized in the occipital areas, whereas neurotypical participants exhibit activation in the fusiform face area (FFA), a brain region heavily engaged in face processing^133^. When seeing inverted faces, SZ patients might use the same strategy as with upright faces, while changing a strategy (from holistic with upright to a serial one with inversion) requires additional efforts in healthy participants. This explanation, although intriguing, seems to be hardly applicable to Face-n-Thing images as they do not contain face cues.

Similar to SZ, the FIE is reduced in neurotypical females as compared to their male peers^62^. However, Face-n-Thing images *most* resembling faces with upright orientation also more often trigger face responses to inverted images in females than in males. By contrast, the images *less* recognizable as faces with upright orientation elicited also more face responses with inversion in SZ individuals than in their neurotypical peers. This implies the distinct origins of the reduced FIE in both cases. Clarification of this issue calls for further experimental work.

At neurophysiological level, face inversion results in an amplitude increase of the N170 ERP (event-related potential) component highly sensitive to faces. This effect is either reduced or absent in SZ patients suggesting abnormal face processing^134–137^. Changes in the N170 component are also associated with lower social functioning^136,138^. The FIE in the electroencephalographic (EEG) gamma-band response in SZ is absent from the electrodes located in the occipital lobe^139^. Taken together, these findings suggest face processing aberrations in SZ may go undetected by commonly used behavioral measures.

### Future directions and limitations

The findings on the brain response to face-like images are sparse. Neuroimaging such as functional magnetic resonance imaging (fMRI)^106,140^ and EEG^61^ reports that the topography and time course of the neural circuits for faces and face-like images appear rather similar. The right FFA is active during perception of noise containing components resembling a face^106^. Perception of both faces and face-like images elicits similar fMRI activation in the occipital cortices, FFA, and inferior temporal regions^140^. EEG shows that 1–4-days-old newborns exhibit activation for face-like stimuli in the right-lateralized network overlapping with the face-processing networks in adults^141^. On the other hand, EEG demonstrates that brain processing of faces and face-like images differ already at earlier stages. Compared with face-like images, faces elicit a larger amplitude and shorter latency of the N170 component of ERP^142,143^.

Only few studies investigated neural correlates of the FIE in SZ, and in none of them face pareidolia images were used. In patients at first episode of SZ, different fMRI activity for upright and inverted faces was found: upright faces activated the face-selective network, whereas inverted faces were associated with isolated activation of the occipital and frontal regions^144^. During presentation of upright faces and non-face images, a significant face-specific effect in the alpha-, beta-, and gamma-band EEG activity was found for controls, whereas SZ patients showed face-specific deficits in the low-frequency beta and gamma band^139^.

Novel insights into the nature of face pareidolia in SZ may be provided by MEG, which can resolve events with millisecond precision^145^. The only MEG study with real faces and face-like stimuli indicates their processing is rather similar: the brain promptly detects the presence of a face^146^. Yet, the superior temporal sulcus (STS) differentiates between real faces and face-like stimuli. These findings should be taken with caution since the study was performed in a small sample (9 participants) and the task was not directed at evaluation of face impression (participants indicated appearance of inverted images).

The present work examined male SZ. The focus on this patient population was set for several reasons: (i) SZ is a male-dominated mental disorder^22,23,25^. (ii) Males and females with SZ possess distinct profiles in social cognition and metacognition^42–44^; (iii) Gender/sex differences are known in face pareidolia^61,82,84^; and (iv) Last but not least, substantial gender differences in the FIE with face-like images have been documented: whereas in males, inversion efficiently prevents face pareidolia, in females, this effect is much less evident^62^. With a desire to attain group homogeneity, only male patients were enrolled in the study. However, female SZ with its own specificity is largely under-investigated, and future psychophysical and brain imaging work should explore gender/sex differences in face tuning. For better understanding of social functioning in SZ, it seems challenging to profile sex differences up to their roots in the brain.

## Data Availability

The data supporting the conclusions of this paper are either included in the paper or will be made available by the authors upon request to any qualified researcher.
